# Particle size distribution and concentration of Intralipid^®^ 20%

**DOI:** 10.1117/1.JBO.31.1.015001

**Published:** 2026-01-23

**Authors:** Mona Shahsavari, Martine Kuiper, Mendel Engelaer, Martin Poinsinet de Sivry-Houle, Ton G. van Leeuwen, Edwin van der Pol

**Affiliations:** a University of Amsterdam, Amsterdam UMC, Biomedical Engineering & Physics, Amsterdam, The Netherlands; b University of Amsterdam, Amsterdam UMC, Laboratory of Experimental Clinical Chemistry, Laboratory Specialized Diagnostics & Research, Department of Laboratory Medicine, Amsterdam, The Netherlands; c Amsterdam Cardiovascular Sciences, Atherosclerosis and Ischemic Syndromes, Amsterdam, The Netherlands; d Cancer Center Amsterdam, Imaging and Biomarkers, Amsterdam, The Netherlands; e VSL, National Metrology Institute, Delft, The Netherlands

**Keywords:** Intralipid^®^, particle size distribution, scattering coefficient, phase function, anisotropy factor, refractive index

## Abstract

**Significance:**

Intralipid^®^ is widely used as a tissue-mimicking phantom due to its optical similarity to biological tissues, i.e., high scattering and low absorption of light. Accurate modeling of the optical behavior of phantoms requires detailed knowledge of the properties of its scatterers.

**Aim:**

We aim to determine the particle size distribution (PSD), concentration, and refractive index (RI) of particles in Intralipid^®^ 20%.

**Approach:**

Cryo-electron microscopy, three different flow cytometers, and microfluidic resistive pulse sensing were used to measure the size distribution and concentration of particles in Intralipid^®^ 20%. To select the most accurate technique, the measured volume fraction of lipid particles was calculated and compared with the expected volume fraction (0.227). The measured size distribution and RI of soybean oil and Dulbecco’s phosphate-buffered saline were used to calculate key optical parameters for the independent, single scattering regime. Calculations were performed with Mie theory across the 400- to 700-nm-wavelength range. Analytical expressions were then fitted to describe the wavelength dependence of each optical parameter.

**Results:**

The PSD, obtained from measurements of more than 10 million particles, spanned from 67 nm to over 2000 nm, with concentration decreasing continuously as diameter increased. The particle volume fraction calculated from the PSD deviated by less than 2% from the expected value, confirming the accuracy of the measurements. Optical parameters calculated from the PSD and RIs, including the scattering coefficient, phase function, anisotropy factor, and reduced scattering coefficient, showed good agreement with values reported in the literature. These parameters were also well described by empirical fits (R>0.97).

**Conclusions:**

The accurate measurement of particle size, concentration, and RI of Intralipid^®^ 20% supports the use of Intralipid^®^ as a reliable tissue-mimicking phantom and calibration sample in optical studies.

## 1 Introduction

Understanding how light interacts with turbid media, such as biological tissue, is fundamental for advancing various medical applications, including medical imaging, laser surgery, and photothermal therapy.^1^ To investigate light–tissue interactions and calibrate optical systems, researchers rely on well-characterized tissue-mimicking phantoms.^2^^–^^4^ Fat emulsions, such as Intralipid^®^, are commonly used as tissue phantoms because they have a high scattering coefficient ($>1\;{\mathrm{mm}}^{-1}$) and a low absorption coefficient ($<0.005\;{\mathrm{mm}}^{-1}$). Scattering can be reduced by dilution, whereas absorption can be increased by adding chromophores. Intralipid^®^ is also non-toxic, affordable, sterile, and widely accessible.^5^^–^^7^

Intralipid^®^ is a polydisperse water–based emulsion containing (1) particles formed by soybean oil droplets encapsulated in a phospholipid monolayer of ∼2.5 to 5 nm thickness and (2) micelle-like particles.^8^ The scattering properties of Intralipid^®^ are influenced by the particle size distribution (PSD) and refractive index (RI) of both the particles and their surrounding liquid.^7^

The PSD of Intralipid^®^ has mainly been measured using transmission electron microscopy (TEM),^9^^,^^10^ showing a distribution that approximates an exponential decay. These PSDs, however, include a few thousand particles and therefore accurately describe particles with a diameter <500  nm only. We noticed that relatively low-abundant particles, i.e., particles with a diameter >700  nm, are unreported. In addition to TEM, dynamic light scattering (DLS) has been used to measure the PSD of Intralipid^®^.^8^^,^^11^^,^^12^ However, as DLS is a bulk technique and as relatively large particles scatter light much more efficiently than small particles, the PSDs of polydisperse samples measured by DLS are biased toward larger particles.^13^ Consequently, DLS is unsuitable to determine the PSD of Intralipid^®^.

The RI of soybean oil droplets, the dominant scattering component of Intralipid^®^, had been measured at 20°C and ranges between 1.56 and 1.44 across the 300 to 2500 nm wavelength range.^14^ The RIs of Dulbecco’s phosphate-buffered saline (DPBS) and water, which can be used as diluents for Intralipid^®^, have also been measured in previous works;^15^^,^^16^ however, these earlier measurements did not include complete uncertainty budgets.

This study aims to measure the full PSD and concentration of Intralipid^®^ 20% using cryo-electron microscopy, flow cytometry, and microfluidic resistive pulse sensing (MRPS). To determine the size of the particles in Intralipid^®^ 20% by calibrated flow cytometers, the RIs of soybean oil and the dispersion medium were traceably measured using the minimum deviation method over the wavelength range of 400 to 700 nm with a complete uncertainty budget.^17^

To select the most accurate technique for PSD measurement of Intralipid^®^ 20%, the volume fraction obtained from the measured PSD was compared with the expected value (0.227). Using the measured PSD and the traceably measured RIs of soybean oil and the dispersion medium, Mie theory was used to calculate key optical parameters, such as the scattering coefficient, phase function, anisotropy factor, and reduced scattering coefficient for Intralipid^®^ 20% diluted in DPBS (1.00% v/v, volume fraction $\phi_{p}=0.00227$), assuming independent, single scattering. To facilitate the practical use of these results, data were fitted with mathematical functions.

## 2 Materials and Methods

### 2.1 Intralipid^®^ 20%

Intralipid^®^ 20% (m/m; Fresenius Kabi Nederland BV, Netherlands) primarily consists of water, soybean oil (200  g/L), and egg lipid (12  g/L). The density of soybean oil according to literature is 0.927  g/mL at 20°C, and that of egg lipid is estimated to be close to 1  g/mL.^7^ Consequently, the expected volume fraction of particles in the emulsion is estimated $\phi_{p\_\text{total}}=0.227$.

To identify the most suitable technique for determining the PSD of Intralipid^®^ 20% (batch 1; lot: 10SH6102, expiry date: January 2025), several techniques were applied and evaluated: cryogenic electron microscopy (cryo-EM), flow cytometry, and MRPS. The technique for which the measured volume fraction was closest to the expectation value was selected and subsequently applied to determine the reference PSD of Intralipid^®^ 20%. Because measurements with the selected technique were conducted after the expiration of Intralipid^®^ batch 1, a second batch (batch 2; lot: 10TA2241, expiry date: June 2025) was analyzed using the selected technique.

To describe the reference PSD, a piecewise function was fitted to the log-transformed, dimensionless particle concentration as a function of dimensionless diameter using linear least-squares regression. A 4th-order polynomial was used in the 57 to 700 nm range, and a linear function in the 700 to 2000 nm range.

The dimensionless form $f(d_{*})=\frac{C(d_{*})}{C_{0}}$ was obtained by expressing the concentrations relative to a reference concentration $C_{0}=1$ (${\mathrm{mL}}^{-1}.{\mathrm{nm}}^{-1}$), where $d_{*}=\frac{d}{d_{0}}$, and the reference size $d_{0}$ is set to 1000 nm. This piecewise function, hereafter referred to as the reference PSD function, is mathematically represented in Eq. (1).

$$\log_{10}\;f(d_{*})=\{\begin{array}{ll} A_{f}\cdot d_{*}^{4}+B_{f}\cdot d_{*}^{3}+C_{f}\cdot d_{*}^{2}+D_{f}\cdot d_{*}+E_{f} & 57<d\leq 700\\ F_{f}\cdot d_{*}+G_{f} & 700<d\leq 2000 \end{array}.$$ (1)

A piecewise function was selected because it provides the best fit to the experimental data across the respective size ranges and ensures accurate representation for subsequent optical modeling.

### 2.2 Cryogenic Electron Microscopy

Intralipid^®^ 20% was diluted 100-fold in DPBS (1.00% v/v) at room temperature. Quantifoil 300 Mesh Cu R2/1 EM grids were glow-discharged in air for 30 s at 0.2 mbar before applying 3  μL of the sample on each grid. The grids were blotted for 3 s at room temperature and 90% humidity using a Leica EM grid plunger and plunge-frozen into a liquid ethane/propane mixture (2:1 v/v) at −196°C. Frozen grids were transferred to a Talos Arctica microscope (Thermo Fisher Scientific) under liquid nitrogen conditions. Images were recorded manually at 900× magnification. Image analysis was performed manually using FIJI (ImageJ v1.54) on a selected image containing a well-distributed range of particle sizes. A total of 1111 lipid particles were annotated, excluding those fully occupying the 2-micron holes, as they were considered artifacts.

### 2.3 Refractive Index Measurement

The RIs of DPBS (21-031-CV, Corning, Corning, NY, USA), soybean oil (S7381, Sigma-Aldrich, St. Louis, MO, USA), and water (TKF7114, Baxter, Deerfield, IL, USA) were measured with metrological traceability at multiple wavelengths using the minimum deviation method.^17^ Measurements were conducted at VSL for seven wavelengths emitted by a mercury-cadmium lamp: 405, 436, 480, 509, 546, 579, and 644 nm at 20±0.1°C. The expanded uncertainty of the measured RI was $1.4\cdot 10^{-5}$ for DPBS and water, and $1.4\cdot 10^{-4}$ for soybean oil. These measurements were used to model the optical dispersion of each liquid using

$$n(\lambda {)}^{2}=A_{n}+B_{n}\cdot \lambda^{2}+\frac{C_{n}}{\lambda^{2}-D_{n}^{2}},$$ (2)

where λ is the wavelength in μm. The function n(λ) represents the RI at wavelength λ, and $A_{n}$, $B_{n}$, $C_{n}$, and $D_{n}$ are the material-specific fitting parameters.

### 2.4 Flow Cytometry Measurements

For flow cytometry measurements, Intralipid^®^ 20% was diluted in DPBS using instrument-specific dilution factors to minimize coincidence and prevent swarm detection.^18^^,^^19^ To ensure homogeneity, the Intralipid^®^ sample was gently tilted prior to dilution, vortexed for 10 s after dilution, and vortexed again for 10 s before the measurement.

For PSD measurements, signals were acquired on each flow cytometer using the side scattering (SSC) detector(s) excited by a 405 nm, 100 mW laser. Details of the measurement settings are summarized in Table 1. For flow cytometer 1 at the detector gain of 10, the 2-min acquisition was used for inter-technique comparison, whereas the 30-min acquisition was used to measure the reference PSD.

**Table 1:** Flow cytometry measurement settings.

Flow cytometer	Dilution factor	Set flow rate (μL/min)	Acquisition time (min)	Trigger detector	Trigger threshold	Detector	Parameter	Voltage/gain
1	648,000	35	2	SSC	1500	SSC	Area	2000
	648,000	35	2 and 30	SSC	580	SSC	Area	10
2	324,000	3.01	2	LALS	24	LALS	Height	367 V
3	810,000	1	6	VSSC-1	200	VSSC-1	Height	49.9
						VSSC-2	Height	30.9

SSC, side scattering; LALS, large-angle light scattering; VSSC, violet side scattering.

The lower limit of detection (LoD) of the SSC detector was defined as five times the standard deviation of a Gaussian fit to the background noise (median set to zero, assuming symmetry about the baseline). Details of the background noise distribution measurements for each flow cytometer are provided in the following subsections.

#### 2.4.1 Flow cytometer 1: Cytek Northern Lights

Flow cytometry measurements were performed using a Northern Lights (Cytek Biosciences, Fremont, CA, USA). The background noise distribution was determined for each data acquisition setting by measuring Milli-Q water while triggering on the V16 detector with a detector gain of 3000 and a threshold level of 1790 arbitrary units (a.u.).

#### 2.4.2 Flow cytometer 2: Apogee A60-micro

Flow cytometry measurements were performed using an A60-Micro flow cytometer (Apogee Flow Systems, Catalonia, Spain). The background noise distribution was determined by measuring Milli-Q water while triggering on the 405Org detector with a detector voltage of 560 V and a threshold level of 22 a.u.

#### 2.4.3 Flow cytometer 3: VDO biotech exoplorer nano-flow cytometer

Measurements were performed using an Exoplorer Nano-flow Cytometer (VDO Biotech; Suzhou, China). For flow cytometers 3, the background noise distributions of both violet SSC detectors (VSSC-1 and VSSC-2) were determined by measuring Milli-Q water while triggering on the VSSC-1 detector with a detector gain of 49.9 and a threshold level of 300 a.u. Because the trigger threshold was set on VSSC-1, only the tail of its background noise distribution above the threshold level was recorded, which was sufficient to determine its LoD.

#### 2.4.4 Light scattering calibration

Rosetta Calibration (v2.05, Exometry, Amsterdam, the Netherlands) was used to relate the arbitrary units of light scattering to the diameter of measured particles.^20^ Therefore, light scattering intensities in arbitrary units were related to the theoretical scattering cross-sections of polystyrene beads with NIST-traceable diameters using Mie theory, thereby taking the wavelength and solid light collection angles of the flow cytometry detectors into account. The calibrated scattering cross sections were in turn related to the particle diameters by Mie theory, assuming that Intralipid^®^ 20% (1) contains droplets with an effective RI equal to soybean oil (at 405 nm) and (2) that the particles are dispersed in pure DPBS. In addition, we assumed that both the monolayer of egg lipids encapsulating the soybean oil and the micelle-like particles have a negligible effect on the PSD and light scattering properties of Intralipid^®^ 20%. This assumption is plausible because (1) the thickness of the phospholipid monolayer is 2 to 3 nm and likely has a similar RI as soybean oil, and (2) with a diameter between 10 and 50 nm,^21^ the diameter of micelles is small compared with the soybean oil droplets and below the detection limit of most of the flow cytometers.

### 2.5 MRPS Measurements

MRPS measurements were conducted using the nCS1 (v0, Spectradyne; USA) along with the nCS1 Viewer software (version 2.5.0.297, Spectradyne). To capture the PSD of Intralipid^®^ 20%, multiple cartridges with overlapping detection ranges were used: C-150 (50 to 150 nm), C-400 (65 to 400 nm), and C-2000 (250 to 2000 nm; two cartridges). To convert voltages to particle diameters, each cartridge is assigned a predefined, mold-specific calibration factor by the manufacturer. Signal discrimination from background noise was performed using cartridge-specific peak filter criteria recommended by the manufacturer. These criteria included thresholds for signal-to-noise ratio (S/N >15 for C-150; S/N >10 for all other cartridges), transit time (<80  μs), and peak symmetry index (0.2<PSI<4.0).

A 1.00% (v/v) Tween-20 solution in DPBS was used as the wetting agent for all MRPS measurements.^22^ This solution was filtered (Nucleopore Track-Etch Membrane, 47 mm, 0.05  μm, Whatman, Maidstone, UK) to remove particles larger than 50 nm. Dilution factors were selected such that the concentration was within the ranges recommended by the manufacturer. Specifically, a dilution factor of $3\cdot 10^{5}$ was used for both the C-150 and C-400 cartridges, whereas dilution factors of $3\cdot 10^{4}$ and $10^{2}$ were applied for the two C-2000 cartridges. The $10^{2}$-fold dilution for one of the C-2000 cartridges was intentionally chosen to measure larger particles, which are present in relatively low concentrations due to the near-exponential decline of the PSD.^10^

To improve accuracy near the detection limit of each cartridge, the mode of each measured PSD was used to approximate the experimental LoD, rather than relying solely on the nominal specifications. The nCS1 Viewer software estimated the analyzed sample volume based on the inverse relationship between the average particle transit time and the volume flow rate through the sensing pore.

### 2.6 Concentration and Particle Size Distribution

To create PSDs, a variable bin width was used in which the width of each bin was defined as 2% of its lower edge, covering a size range from 40 to 2000 nm. To combine PSDs acquired from different MRPS cartridges or by different flow cytometry settings, we used a weighted average. Weights were assigned based on the number of particles detected in each bin, ensuring that the combined PSD accurately represented the relative contributions of each individual measurement. To ensure that the Poisson error remained <50% per bin, bins with a concentration based on <5 particle counts were excluded. The stock concentration of Intralipid^®^ 20% was determined by multiplying the measured size distribution of diluted samples by the corresponding dilution factor.

### 2.7 Volume Fraction of Particles

The volume fraction was calculated by multiplying the particle concentration in each bin by the corresponding particle volume and summing over all bins. Each measurement technique detects particles within a limited size range and therefore measures a part of the full PSD of Intralipid^®^ 20%. As a result, the measured volume fraction underestimates the total volume fraction of particles, making direct comparisons with the expectation value ($\phi_{p\_\text{total}}=0.227$) unreliable. To address this problem, we used the reference PSD function described in Sec. 2.1, which covers the broadest size range (57 to 2000 nm) over which the PSD of Intralipid^®^ 20% was determined. This function was assumed to represent the full PSD of Intralipid^®^ 20%.

For each technique, we applied a correction factor to account for the fraction of the PSD that was undetected. The correction factor was computed by dividing the volume fraction derived from the reference PSD function (i.e., across the full size range) by the partial volume fraction derived from the same function, but constrained to the detection range of the technique. By multiplying the measured volume fraction by the correction factor, we accounted for the limited detection range of each technique. This method allowed for meaningful comparisons between the measured and expected volume fractions.

### 2.8 Optical Properties

The scattering coefficient ($\mu_{s}$), phase function (P(θ)), anisotropy factor (g), and reduced scattering coefficient ($\mu_{s}^{\prime }$) for Intralipid^®^ 20% diluted in DPBS with a volume fraction of $\phi_{p}=0.00227$ were calculated using Mie theory. Calculations were performed over the wavelength range of 400 to 700 nm using the measured RIs of DPBS and soybean oil as input parameters. The scattering coefficient density was computed using both the reference PSD and its fitted function described in Sec. 2.1, whereas all other optical properties were derived solely from the reference PSD.

To model the optical properties, we assumed independent, single scattering. This assumption is valid for scattering emulsions with a volume fraction $\phi_{p}\leq 0.02$.^23^^–^^25^ Under this assumption, separate Mie theory calculations were performed for each bin in the distribution (see Sec. 2.6 on binning).

The Mie scattering calculations were performed using the PyMieScatt Python package^26^ and that only the real part of the RI was used, because the reported absorption of soybean oil is negligible^14^ and does not measurably affect the calculated optical properties.

The scattering coefficient density, defined as the average number of scattering events per unit length for particles in bin b, is calculated as

$$\mu_{s}^{\left(b\right)}=\sigma^{\left(b\right)}\cdot C^{\left(b\right)},$$ (3)

where $\sigma^{\left(b\right)}$ and $C^{\left(b\right)}$ are the scattering cross section and concentration, respectively, for particles in bin b. The total scattering coefficient ($\mu_{s}$) of the solution is obtained by summing the scattering coefficient densities over the bins

$$\mu_{s}=\sum_{b}\mu_{s}^{\left(b\right)}.$$ (4)

The overall phase function, P(θ), which describes the angular distribution of scattered light, was computed as

$$P(\theta )=\underset{b}{\sum }\frac{P^{\left(b\right)}(\theta )\cdot C^{\left(b\right)}}{C},$$ (5)

where θ is the angle between the direction of the incident light beam and the direction of the scattered light, $P^{\left(b\right)}(\theta )$ is the phase function, and $C^{\left(b\right)}$ is the corresponding concentration of particles in bin b. The total particle concentration in the solution is given by $C=\sum_{b}C^{\left(b\right)}$. Thus, P(θ) represents a concentration-weighted average of the individual phase functions across all particle bins. Using P(θ), the anisotropy factor (g) is computed as

$$g=\frac{\int_{0}^{\pi }P(\theta )\cdot \cos(\theta )\cdot \sin(\theta )d\theta }{\int_{0}^{\pi }P(\theta )\cdot \sin(\theta )d\theta }.$$ (6)

Knowing the scattering coefficient and anisotropy factor, the reduced scattering coefficient can be determined using

$$\mu_{s}^{\prime }=(1-g)\cdot \mu_{s}.$$ (7)

The calculated optical properties were then compared with empirical functions previously reported by Michels et al.^7^ and Aernouts et al.^23^

### 2.9 Mathematical Representation of Optical Properties

For all wavelength-dependent expressions in the range of 400 to 700 nm, we introduced the normalized wavelength variable $\lambda^{*}=\frac{\lambda }{\lambda_{0}}$ with $\lambda_{0}=500\;\mathrm{nm}$.

The scattering coefficient ($\mu_{s}$) was described by the power-law function

$$\mu_{s}(\lambda ,\phi_{p})=\alpha \cdot (\lambda^{*}{)}^{-\beta }\cdot (\frac{\phi_{p}}{\phi_{p\_\text{total}}}),$$ (8)

where α and β are the fitting parameters.

To provide a mathematical expression for the phase function P(θ,λ) across the full scattering angle (θ) range, a function was fitted to $\log_{10}(P(\eta ,\lambda^{*}))$, as a function of η=−cos θ, and the normalized wavelength. The fitted model is given by

$$P(\eta ,\lambda^{*})=\frac{A_{P}\eta^{3}+B_{P}\eta^{2}+C_{P}\eta +D_{P}+E_{P}\lambda^{*}}{F_{P}\eta^{3}+G_{P}\eta +H_{P}\lambda^{*}},$$ (9)

where η=−cos θ and $A_{P}$, $B_{P}$, $C_{P}$, $D_{P}$, $E_{P}$, $F_{P}$, $G_{P}$, and $H_{P}$ are the fitting parameters.

The anisotropy factor (g) was expressed by a linear function as

$$g(\lambda )=\zeta +\gamma \cdot (\lambda^{*}),$$ (10)

where ζ and γ are the fitting parameters.

The reduced scattering coefficient ($\mu_{s}^{\prime }$) was described using a power-law model

$$\mu_{s}^{\prime }(\lambda ,\phi_{p})=\alpha^{\prime }\cdot (\lambda^{*}{)}^{-\beta^{\prime }}\cdot (\frac{\phi_{p}}{\phi_{p\_\text{total}}}),$$ (11)

where $\alpha^{\prime }$ and $\beta^{\prime }$ are the fitting parameters.

The fitting parameters in Eqs. (8)–(11) were obtained by fitting the corresponding functions to the calculated optical properties using linear least-squares regression.

## 3 Results

### 3.1 Morphology and Particle Size Distribution of Intralipid^®^ 20% Measured with Cryogenic Electron Microscopy

**Fig. 1:**
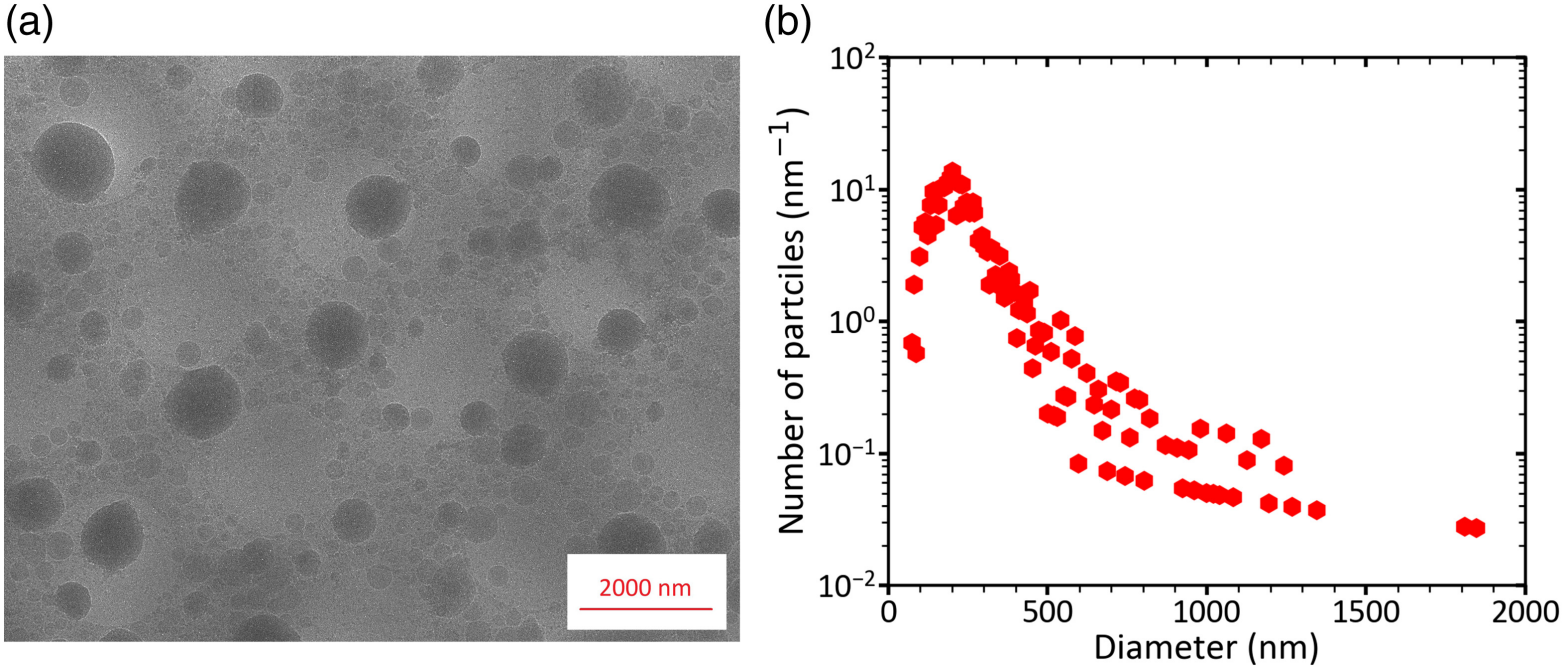
Cryogenic electron microscopy (cryo-EM) of 1% (v/v) dilution of Intralipid^®^ 20% in Dulbecco’s phosphate-buffered saline. (a) Representative cryo-EM image of Intralipid^®^ 20%. (b) Distribution of particle counts as a function of diameter. Bin widths are defined in the Methods.

Figure 1(a) shows a cryo-EM image of particles in a 1.00% (v/v) dilution of Intralipid^®^ 20% in DPBS. Due to the limited contrast and rather small magnification, no structural information regarding particle membranes could be obtained from the images (see Fig. S1 in the Supplementary Material). Figure 1(b) shows the distribution of particle counts as a function of diameter for a 1% (v/v) dilution of Intralipid^®^ 20%. Based on results obtained with other techniques,^9^^,^^10^ the mode of the PSD at ∼200  nm is likely an artefact. Under ideal conditions, cryo-EM is capable of detecting particles <200  nm. However, in our measurements, the detection efficiency for particles <200  nm was probably reduced due to low image contrast. We attribute this loss of contrast to a thick layer of vitrified water, which arises from the presence of relatively large particles in Intralipid^®^ 20%. For example, the data reveal the presence of particles with diameters between 1 and 2  μm.

### 3.2 Refractive Index Measurements

**Fig. 2:**
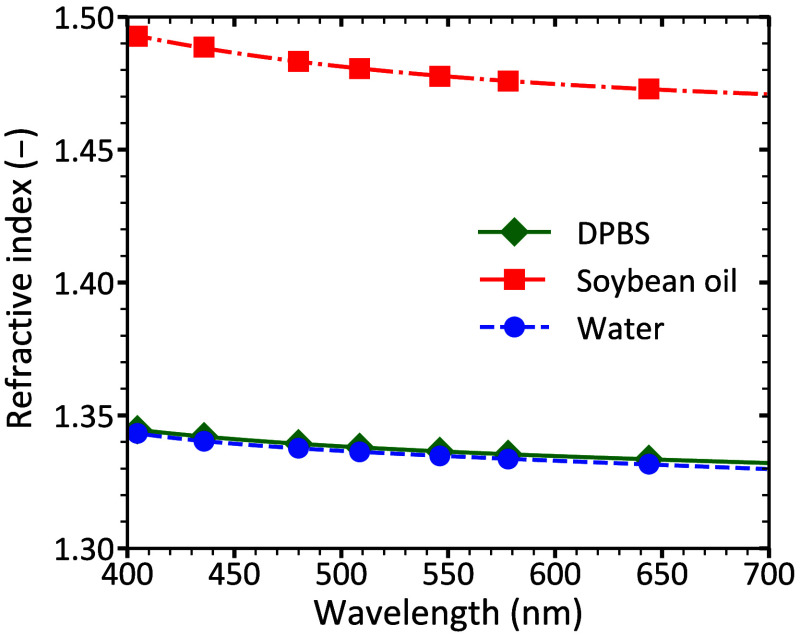
Measured refractive indices of Dulbecco’s phosphate-buffered saline (DPBS; diamonds), soybean oil (squares), and water (circles) at wavelengths 405, 436, 480, 509, 546, 579, and 644 nm, along with their corresponding fits based on Eq. (2). The fits are shown as a green solid line for DPBS, a red dash-dot line for soybean oil, and a blue dashed line for water.

As flow cytometry–based sizing and light scattering calculations require the RI of particles and their medium, we performed RI measurements with a minimum angle of deviation setup. Figure 2 shows the experimentally measured RIs of DPBS, soybean oil, and water at seven discrete wavelengths: 405, 436, 480, 509, 546, 579, and 644 nm. The corresponding numerical values are summarized in Table 2.

**Table 2:** Experimentally measured refractive indices of Dulbecco’s phosphate-buffered saline (DPBS), soybean oil, and water at selected wavelengths.

Wavelength (nm)	405	436	480	509	546	579	644
DPBS	1.344599	1.341988	1.339333	1.337966	1.336453	1.335331	1.333467
Soybean oil	1.492682	1.488585	1.483253	1.480541	1.477609	1.475855	1.472850
Water	1.343266	1.340213	1.337721	1.336331	1.334657	1.333649	1.331547

The dispersion relations obtained by fitting Eq. (2) across the 400 to 700 nm range are also shown in Fig. 2. All fitted models yielded coefficients of determination ($R^{2}$) greater than 0.999, and the corresponding fit parameters are provided in Table 3.

**Table 3:** Fitted parameters of Eq. (2) for Dulbecco’s phosphate-buffered saline (DPBS), soybean oil, and water.

Liquid	$A_{n}$ (−)	$B_{n}$ ($\mu m^{-2}$)	$C_{n}$ ($\mu m^{2}$)	$D_{n}$ (μm)
DPBS	1.77611183	$-2.32547738\cdot 10^{-2}$	$4.33441896\cdot 10^{-3}$	$2.05080232\cdot 10^{-1}$
Soybean oil	2.12615032	$6.84946354\cdot 10^{-3}$	$1.66137788\cdot 10^{-2}$	$2.35999596\cdot 10^{-3}$
Water	1.78685001	$-4.73643733\cdot 10^{-2}$	$1.85725893\cdot 10^{-3}$	$3.00086689\cdot 10^{-1}$

Parameters are valid over the 400 to 700 nm wavelength range.

### 3.3 Calibration of Flow Cytometry Measurements

**Fig. 3:**
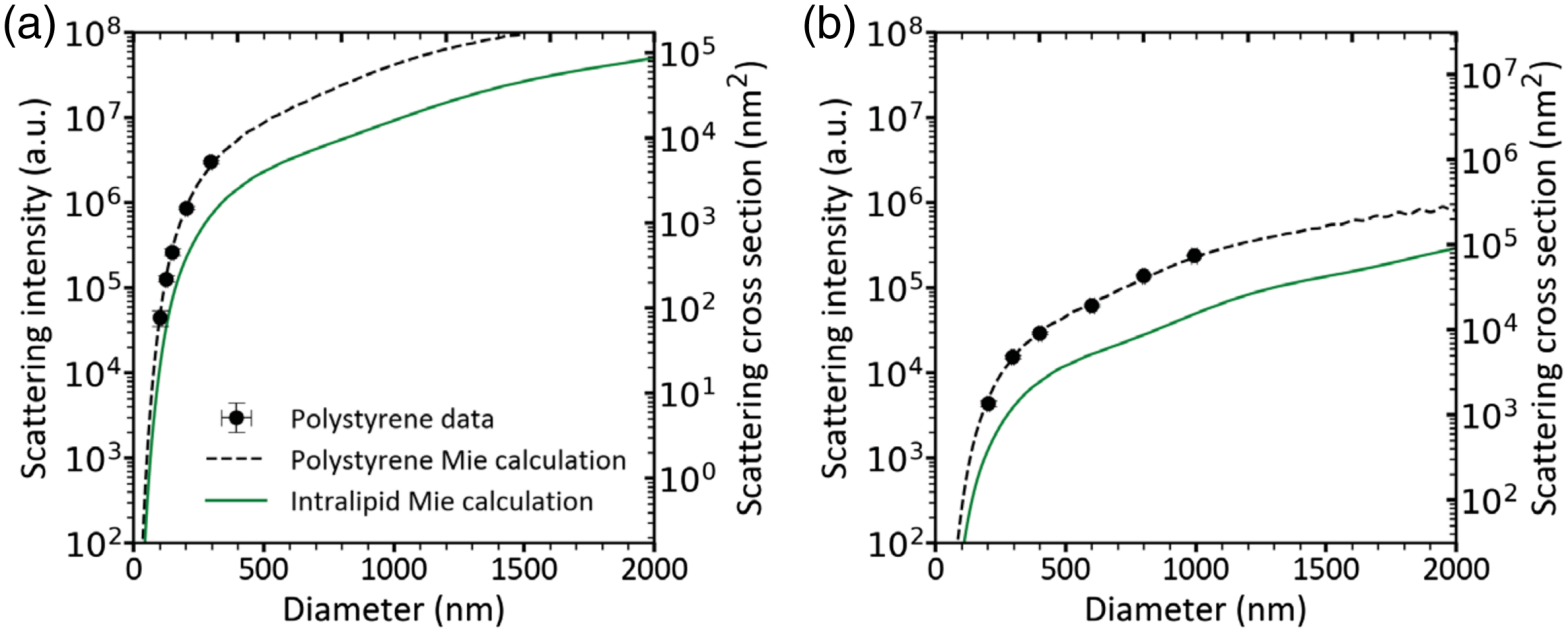
Side scatter calibration of flow cytometer 1 (Northern Lights) using Rosetta calibration.^20^ The black circle marker shows the median side scattering (SSC) intensities (left axis, arbitrary units) for polystyrene bead populations versus their specified mean diameters. Error bars indicate the standard deviation of the measured signals and the specified diameters. The dashed black line shows the theoretical side scattering cross sections (right axis in $nm^{2}$) calculated using Mie theory for polystyrene beads (RI = 1.633 at 405 nm) in water (RI = 1.3433). A scalar was obtained by least square fitting to match the side scattering cross sections to the measured SSC intensities, enabling conversion from arbitrary units to physical scattering units. This scalar was subsequently used to relate SSC signals of Intralipid^®^ 20% to particle diameters. Intralipid^®^ particles were modeled as soybean oil droplets (RI = 1.4927) suspended in DPBS (RI = 1.3446), under identical optical settings (green solid line). (a) Gain: 2000; Scalar: $1.74\cdot 10^{-3}$. (b) Gain: 10; Scalar: $3.09\cdot 10^{-1}$.

To explain the conversion of SSC signals in arbitrary units to particle diameters using Rosetta calibration,^20^ the calibration procedure applied to flow cytometer 1 is shown in Fig. 3. Figures 3(a) and 3(b) show the calibration for a detector gain of 2000 and 10, respectively.

To establish the calibration curve, a mixture of ten polystyrene bead populations with NIST-traceable diameters ranging from 70 to 994 nm was used. Using Mie theory, the optical configuration of Flow Cytometer 1 as well as the RIs of polystyrene (1.633) and water (1.3433) at a wavelength of 405 nm were used to calculate the side scattering cross section for each bead size. The data were then fitted onto the theoretical side scattering cross sections by least square fitting, resulting in a scalar enabling the conversion of SSC signals (a.u.) into side scattering cross sections (${\mathrm{nm}}^{2}$). The scalar was subsequently applied to SSC measurements of Intralipid^®^ 20% obtained under identical instrument settings. Intralipid^®^ particles were modeled as soybean oil droplets suspended in DPBS, with a RI of 1.4927. As shown by the green solid line in Fig. 3, the resulting calibration curve can be used to derive the diameter of particles in Intralipid^®^ 20% for any given SSC signal.

### 3.4 Concentration and Particle Size Distribution of Intralipid^®^ 20%

**Fig. 4:**
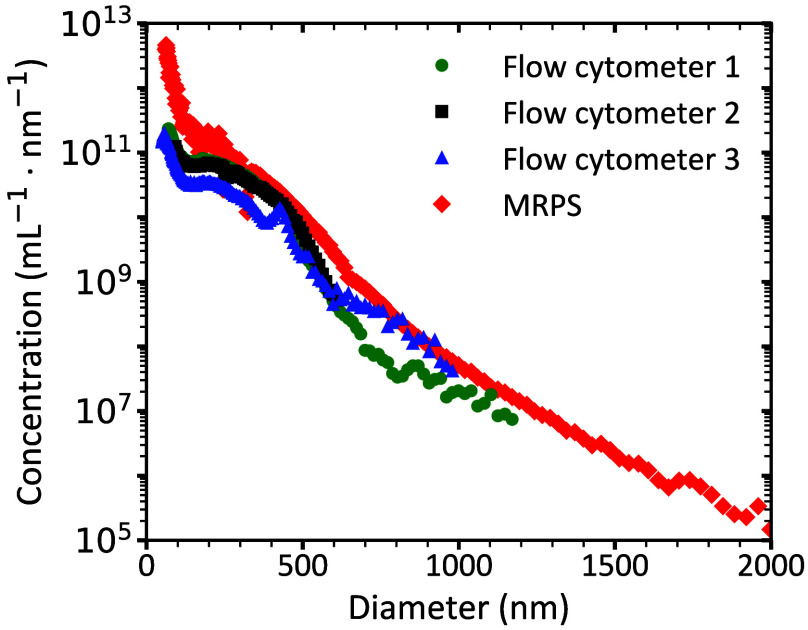
Concentration versus diameter of particles in stock Intralipid^®^ 20% measured by flow cytometer 1 (Northern Lights; circles), flow cytometer 2 (A60-Micro; squares), flow cytometer 3 (Exoplorer Nano-flow cytometer; triangles), and microfluidic resistive pulse sensing (MRPS; nCS1; diamonds). To report the stock concentration of Intralipid^®^ 20%, we multiplied the measured PSD of diluted samples, as described in the Methods Sec. 2.6, with the dilution factor. For calibrating flow cytometer results, particles in Intralipid^®^ 20% were modeled as soybean oil droplets (refractive index = 1.4927) in Dulbecco’s phosphate-buffered saline (refractive index = 1.3446).

Figure 4 shows the concentration and PSD of stock Intralipid^®^ 20% measured using flow cytometer 1, 2, 3, and MRPS within the size range of 68 to 1200, 90 to 608, 48 to 1000, and 60 to 2000 nm, respectively.

Given the large dynamic range for both concentration ($∼1:10^{8}$) and diameter (1:42), the data are in good agreement. As expected, we measured a near-exponential decline of the particle concentration with increasing diameter for all techniques. The curves show a shoulder between 200 and 600 nm. At 430 nm, the data of flow cytometer 3 show a peak that is not representative of the actual PSD of Intralipid^®^ 20%. We attribute this peak to flattening of the scatter-to-diameter relationship around 430 nm, which leads to the measurement of similar scattering intensities for particles of varying sizes and thus to an artificial peak in the PSD.^27^ Despite this artifact, flow cytometer 3 has the highest sensitivity among the techniques tested and shows a peak at 57 nm for the PSD of Intralipid^®^ 20%.

To assess the quantitative accuracy of each technique, the volume fraction was derived from the measured PSDs and compared with the expected value of $\phi_{p\_\text{total}}=0.227(v/v)$. The measured volume fraction of particles in Intralipid^®^ 20% was 0.213, 0.202, 0.126, and 0.412 (v/v) for flow cytometers 1, 2, and 3, and MRPS, respectively. After correcting for the differences in the measurable size range, as explained in Sec. 2.7, the adjusted volume fractions were 0.215, 0.212, 0.127, and 0.414 (v/v), corresponding to relative differences of 5.3%, 6.6%, 44%, and 82% from the expected value.

Thus, flow cytometers 1 and 2 show the highest accuracy in measuring the volume fraction. As flow cytometer 1 provides a broader measurable size range than flow cytometer 2, we decided that flow cytometer 1 was most suitable for determining the reference PSD of Intralipid^®^ 20%.

### 3.5 Reference Particle Size Distribution of Intralipid^®^ 20%

**Fig. 5:**
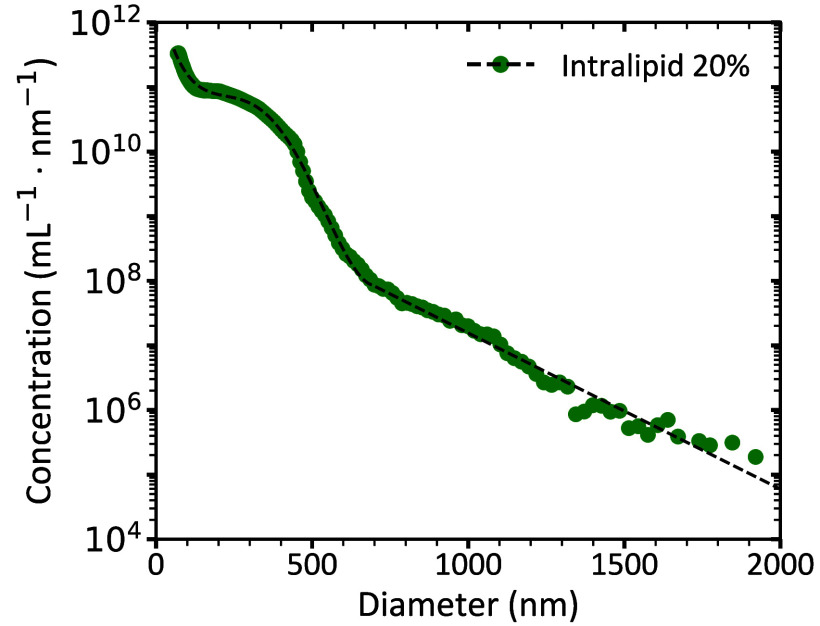
Concentration versus diameter of particles in Intralipid^®^ 20% measured (circles) by flow cytometer 1 (Northern Lights). To report the stock concentration of Intralipid^®^ 20%, we multiplied the measured PSD of diluted samples, as described in the Methods Sec. 2.6, with the dilution factor. For calibration of flow cytometry results, particles in Intralipid^®^ 20% were modeled as soybean oil droplets (refractive index = 1.4927) in Dulbecco’s phosphate-buffered saline (refractive index = 1.3446). The black dashed line shows the fitted function described in Eq. (7), with a coefficient of determination ($R^{2}$) of 0.970. 73 particles with a diameter >2000  nm were detected but excluded during fitting based on their Poisson error (see Methods Sec. 2.6).

Figure 5 shows the reference PSD of stock Intralipid^®^ 20% measured with flow cytometer 1 between 68 and 2000 nm. Similar to Fig. 4, we measured a near-exponential decay of the particle concentration with increasing diameter and a shoulder between 200 and 600 nm.

To enable further analysis, the PSD was mathematically described by a piecewise function as described in Eq. (1). The fit resulted in coefficients of determination $R^{2}>0.970$. The fit coefficients are provided in Table 4.

**Table 4:** Dimensionless coefficients for the fitted function described in Eq. (1).

$A_{f}$	$B_{f}$	$C_{f}$	$D_{f}$	$E_{f}$	$F_{f}$	$G_{f}$
151.5	−231.2	109.9	−22.18	12.53	−2.417	9.609

Although the measured PSD from flow cytometer 1 begins at 68 nm, the PSD of flow cytometer 3 (Sec. 3.4) showed that the true peak of the distribution is at 57 nm. To improve the representation of the full PSD, the fitted function was extended to start at 57 nm. The calculated volume fractions of particles based on the measured and fitted PSDs were 0.223 and 0.218 (v/v), respectively. These values differ by only 2% and 4% from the expected value of 0.227 (v/v), thereby showing high accuracy.

### 3.6 Scattering Coefficient

**Fig. 6:**
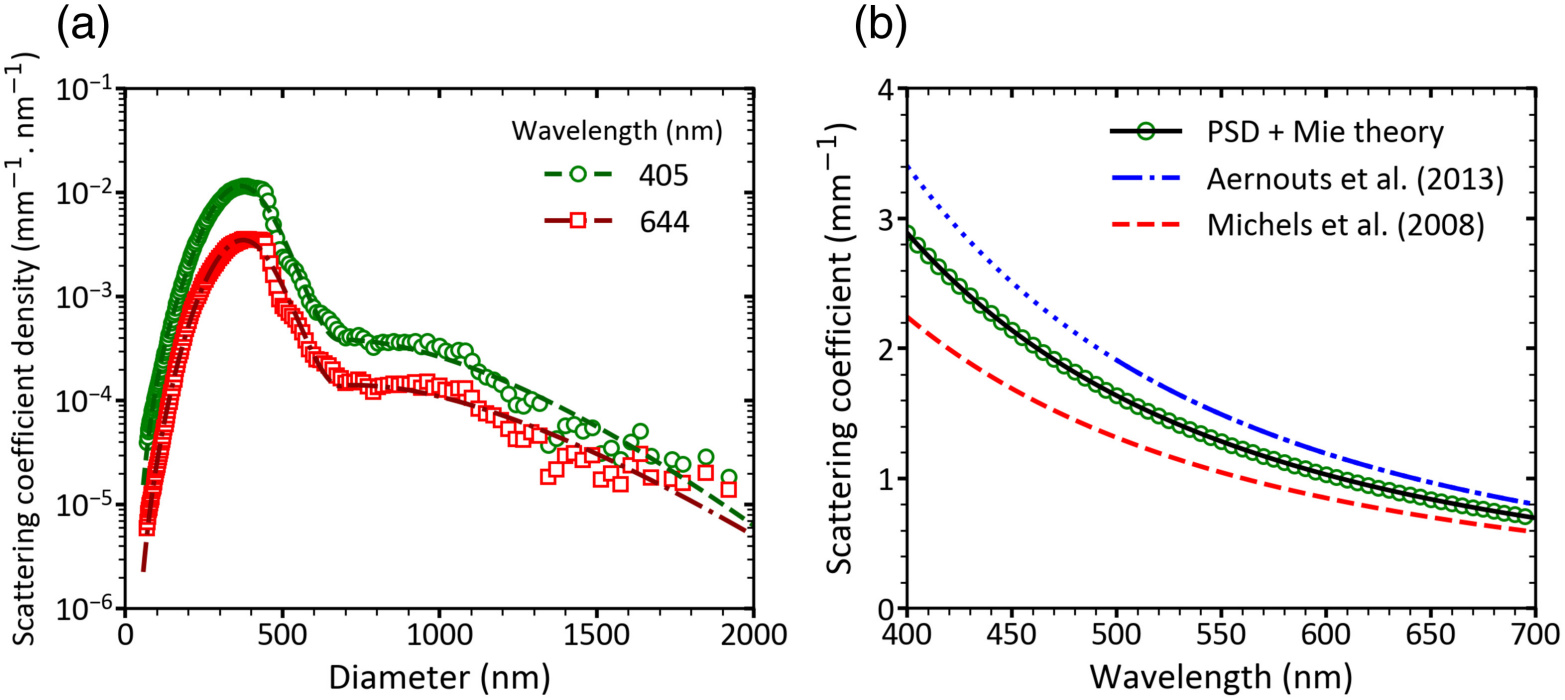
Scattering coefficient of Intralipid^®^ 20% diluted in Dulbecco’s phosphate-buffered saline to a volume fraction $\phi_{p}=0.00227$. (a) Scattering coefficient density per bin as a function of particle diameter. Circles and squares correspond to wavelengths of 405 and 644 nm, respectively, calculated from the reference particle size distribution (PSD). Solid and dashed lines show the corresponding results based on the reference PSD function [Eq. (1)]. (b) Scattering coefficient $\mu_{s}$ as a function of wavelength, calculated at 5 nm intervals across the 400- to 700-nm spectral range. Circles show Mie theory results based on the reference PSD. The solid black line is a power-law fit [Eq. (8)] incorporating the volume fraction. For comparison, literature data calculated for the same volume fraction from Aernouts et al.^23^ and Michels et al.^7^ are shown as dashed and dash-dotted lines, respectively. Data from Aernouts are shown as a dash-dotted line in its valid range (500 to 700 nm) and as a dotted line below 500 nm, where it is extrapolated.

The calculated scattering coefficient of Intralipid^®^ 20% diluted in DPBS (1.00% v/v, $\phi_{p}=0.00227$) is shown in Fig. 6. Figure 6(a) shows the scattering coefficient density at 405 nm and 644 nm based on the reference PSD and the reference PSD function of Intralipid^®^ 20%. For both wavelengths, the scattering coefficient density peaks at ∼380  nm.

Figure 6(b) shows the total scattering coefficient ($\mu_{s}$) as a function of wavelength from 400 to 700 nm for $\phi_{p}=0.00227$. Calculated values based on the reference PSD show good agreement with data from the literature,^7^^,^^23^ with relative deviations below 25%. Fitting $\mu_{s}$ with the power-law function described in Eq. (8), leads to $\alpha =163.7\;{\mathrm{mm}}^{-1}$ and β=2.546 with a coefficient of determination $R^{2}>0.996$. Please note that Eq. (8) is only valid in the independent, single scattering regime ($\phi_{p}<0.02$).

### 3.7 Phase Function

**Fig. 7:**
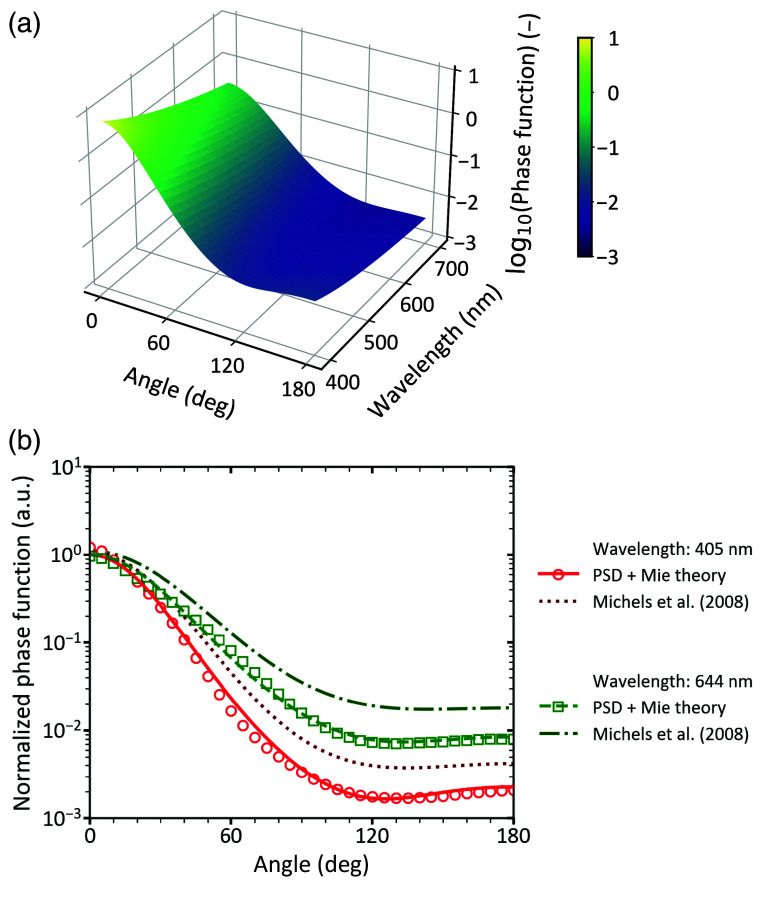
Phase function of Intralipid^®^ 20% assuming independent, single scattering (volume fraction< 0.02). (a) Logarithm of the phase function as described by Eq. (9), plotted as a function of scattering angle across the wavelength range of 400 to 700 nm. (b) Normalized phase function as a function of scattering angle. The maximum value was normalized to 1. Circle and square markers represent values at 405 and 644 nm, respectively, computed using Mie theory based on the reference particle size distribution. Fitted phase functions based on Eq. (9) are shown as solid and dashed lines for 405 and 644 nm, respectively. For comparison, phase functions derived from the measurements of Michels et al.^7^ are shown as dotted and dash-dotted lines.

The phase function of Intralipid^®^ 20% was computed using Mie theory based on the reference PSD, assuming independent, single scattering. The function given in Eq. (9) was fitted to the calculated phase function, yielding a coefficient of determination $R^{2}>0.997$ for all wavelengths ranging from 400 to 700 nm. The fitted parameters are summarized in Table 5. Figure 7(a) shows a 3D surface plot of Eq. (9), showing the logarithm of the phase function as a function of scattering angle and wavelength in the range of 400 to 700 nm.

**Table 5:** Dimensionless coefficients for the fitted function described in Eq. (9).

$A_{P}$	$B_{P}$	$C_{P}$	$D_{P}$	$E_{P}$	$F_{P}$	$G_{P}$	$H_{P}$
−1520.6	2240.1	−20.513	1836.6	−5034.5	399.48	−934.27	1696.6

The proposed phase function model was compared with an empirical expression previously introduced by Michels et al.^7^ Because the formulation of Michels et al.^7^ is normalized to a maximum value of one, all computed phase functions were similarly normalized for consistency. Figure 7(b) shows the computed normalized phase functions and corresponding fits for two representative wavelengths (405 and 644 nm), using both the proposed model and the formulation of Michels et al.^7^

### 3.8 Anisotropy Factor and Reduced Scattering Coefficient

**Fig. 8:**
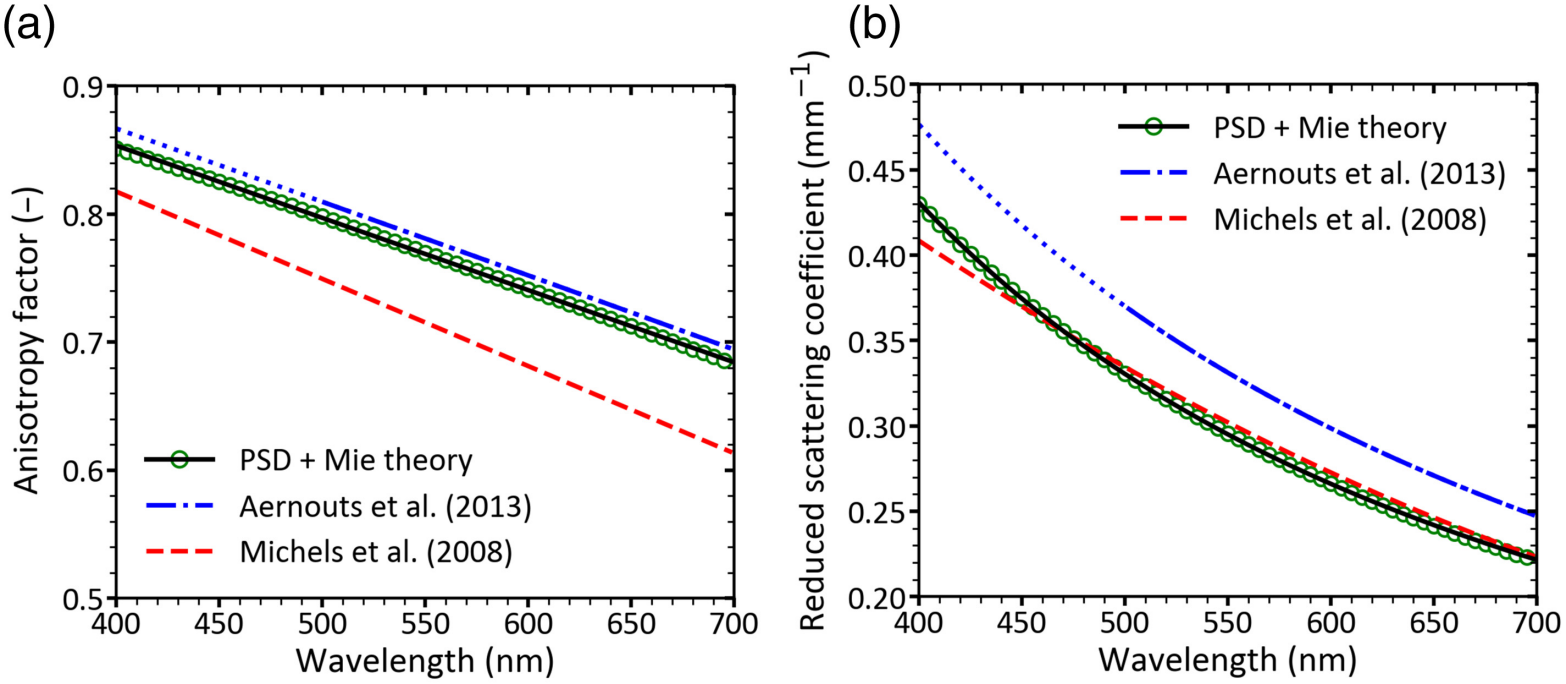
(a) Anisotropy factor and (b) reduced scattering coefficient of Intralipid^®^ 20% diluted in Dulbecco’s phosphate-buffered saline for a volume fraction $\phi_{p}=0.00227$. Data are presented as a function of wavelength, calculated at 5 nm intervals over the 400 to 700 nm spectral range. Green hollow circle markers show Mie theory results based on the reference PSD. The solid black lines indicate fitted functions to the calculated data, as described in Eqs. (10) and (11). For comparison, literature values from Aernouts et al.^23^ and Michels et al.^7^ are shown as dashed and dash-dotted lines, respectively. Data from Aernouts are shown as a dash-dotted line in its valid range (500 to 700 nm) and as a dotted line below 500 nm, where it is extrapolated.

Figure 8(a) shows the anisotropy factor (g) determined using Mie theory and the reference PSD of Intralipid^®^ 20%, assuming independent, single scattering. These results are compared with previously reported values in the literature.^7^^,^^23^ The obtained values of g in this study fall between previously reported values, with a relative difference of <8%. A linear dependence of g on wavelength is observed. Fitting the obtained values of g with the function described in Eq. (10), leads to ζ=1.079 and γ=−0.282 with a coefficient of determination greater than $R^{2}>0.996$.

Figure 8(b) shows the reduced scattering coefficient ($\mu_{s}^{\prime }$) of Intralipid^®^ 20% diluted in DPBS (1.00% v/v, $\phi_{p}=0.00227$) as a function of wavelength between 400 and 700 nm. Calculated values (green hollow circles) based on the reference PSD show good agreement with literature data,^7^^,^^23^ with relative differences below 11%. Fitting $\mu_{s}^{\prime }$ with the power law function described in Eq. (11), leads to $\alpha^{\prime }=33.04\;{\mathrm{mm}}^{-1}$ and $\beta^{\prime }=1.189$ with a coefficient of determination greater than $R^{2}>0.999$. Please note that Eq. (11) is only valid in the independent, single scattering regime ($\phi_{p}<0.02$).

## 4 Discussion and Conclusion

In this study, we measured the size and concentration of >10 million particles in Intralipid^®^ 20% with flow cytometry. We obtained a PSD ranging from 68 nm to 2000 nm, which is the most comprehensive PSD of Intralipid^®^ reported to date. In addition, we determined the RI of DPBS, soybean oil, and water across the 400 to 700 nm wavelength range with an unprecedented uncertainty of $1.4\cdot 10^{-5}$ for DPBS and water, and $1.4\cdot 10^{-4}$ for soybean oil. Combining these data, we determined the scattering coefficient, phase function, anisotropy factor, and the reduced scattering coefficient of diluted Intralipid^®^ (= 0.00227) and found good agreement with published values. To support practical use of our findings, all results were fitted with empirical equations, yielding coefficients of determination >0.97.

To select the optimal sizing technique, we measured the PSD of Intralipid^®^ 20% with cryo-EM, three flow cytometers, and MRPS and compared the measured volume fractions with the expected value of 0.227 (v/v). Cryo-EM provided visual confirmation of the broad size range of Intralipid^®^ particles, spanning 75 nm to >2000  nm. In contrast to the PSDs reported by other groups,^9^^,^^10^ the cryo-EM PSD decreases below 200 nm. Although transmission EM can have sub-nm resolution, we attribute this discrepancy to reduced image contrast of relatively small particles (see Fig. S1 in the Supplementary Material), possibly due to local thickening of the ice layer caused by the presence of relatively large particles.

Within their respective detection ranges, the PSD of Intralipid^®^ 20% measured with the three flow cytometers and MRPS are in good agreement with each other (Fig. 4) and with literature values.^9^^,^^10^ Flow cytometers 1 and 2 yielded volume fractions closest to the expected value, with deviations under 6%. Because flow cytometer 1 also covered the broadest measurable size range (68 to 2000 nm), it was selected as the most suitable instrument for measuring the reference PSD of Intralipid^®^ 20%. The reference PSD was fitted with Eq. (1), showing excellent agreement with the data ($R^{2}>0.970$). The volume fraction derived from the fit (0.218 (v/v)) deviated by 4% from the expected value, supporting the accuracy of the fitted model.

Based on the reference PSD and RI data, optical properties, including the scattering coefficient ($\mu_{s}$), phase function (P(θ)), anisotropy factor (g), and reduced scattering coefficient ($\mu_{s}^{\prime }$), were determined for the independent, single scattering regime over the 400 to 700 nm wavelength range. Analytical expressions were then fitted to these calculated properties to facilitate knowledge update.

The total scattering coefficient followed a power-law dependence on wavelength, consistent with literature values, with deviations below 25%. The anisotropy factor decreased linearly with wavelength, and the reduced scattering coefficient followed a power-law trend. Both parameters were in good agreement with previously reported data, with deviations <8% for g and 11% for $\mu_{s}^{\prime }$, further confirming the accuracy of the reference PSD.

### 4.1 Limitations of the Study

To determine particle sizes in Intralipid^®^ 20% scattering signals measured by flow cytometry, we modeled the particles as spherical soybean-oil droplets encapsulated by a single phospholipid monolayer and suspended in DPBS. However, several studies suggest that Intralipid^®^ particles can possess multilayered phospholipid shells and internal water inclusions.^7^^,^^28^ Such structural complexity may compromise the validity of the single-layer droplet model and potentially affect the resulting size estimates. In addition, as the RI of egg-derived lipids is not well-characterized, it is commonly approximated by that of soybean oil. If the RI of egg lipids were known, a more accurate core–shell model could be applied to better describe the scattering behavior of Intralipid^®^ particles.

We assumed that below a volume fraction of 0.02, there is independent, single scattering, as has been confirmed in mono-disperse samples with silica beads.^24^^,^^25^ This assumption is probably also valid for a heterogeneous sample like Intralipid^®^, as shown by di Ninni et al.^29^ and Zaccanti et al.^30^ However, Driver et al.^31^ showed that the effective attenuation coefficient did not behave linearly for volume fractions below 0.01. Further theoretical investigations, similar to Raju and Unni^8^ but based on our reference PSD, might be needed to substantiate the dependent scattering effects measured by Speets et al.^32^ and Aernouts et al. ^33^

The scattering properties of stirred Intralipid^®^ are stable, although prolonged storage can lead to the development of a surface layer with elevated scattering, causing non-uniform optical properties. For example, the relative systematic error in the reduced scattering coefficient was observed to gradually increase from 7% immediately after stirring to 25% after 1 h.^34^ This problem can be mitigated through stirring, which restores uniform scattering properties without changing its optical characteristics.^34^

All optical properties were calculated assuming DPBS as the diluent. The mean relative difference between the scattering coefficients obtained using water and DPBS as the diluent was 3%. Corresponding differences were 1% for the phase function, 0.07% for the anisotropy factor, and 3% for the reduced scattering coefficient, showing that the effect of the diluent on the calculated optical properties is negligible. However, DPBS is preferred over water because it better mimics physiological conditions and maintains osmotic balance, which helps preserve the structural stability of lipid-based emulsions such as Intralipid^®^ during dilution and measurement.

Figure S2 in the Supplementary Material shows the PSDs of two batches of Intralipid^®^ 20%, as well as measurements of the same batch obtained before and three months after its expiration date using flow cytometer 1. The PSDs closely overlap, showing comparable size distributions across batches and storage conditions. The measured particle concentrations, however, differed, with a mean relative difference of 19% between the two batches and 19% between measurements performed before and after expiration. To evaluate the impact of these differences on the calculated optical properties, scattering coefficients were calculated over the wavelength range of 400 to 700 nm using each PSD. The corresponding mean relative differences in the scattering coefficient were 7% for the two-batch comparison and 5% for the before-versus-after-expiration comparison. These measurements were performed over a six-month period using different acquisition times (2 and 10 min for batch 1 and 30 min for batch 2), which may partly contribute to the observed concentration variability. A more systematic investigation of aging, batch-to-batch variability, and inter-brand differences under identical experimental conditions and shorter time frames would be valuable; however, this falls outside the scope of the present work and will be addressed in future studies.

In conclusion, the integration of precise RI measurements, broad PSD analysis, and Mie-theory modeling enabled accurate determination of the optical properties of Intralipid^®^ 20% in the independent, single scattering regime. The proposed models support the use of Intralipid^®^ 20% as a reliable tissue-mimicking phantom and calibration sample^35^ in optical studies. Future work will investigate the effects of long-term storage without stirring, inter-brand variation, different Intralipid^®^ concentrations (e.g., 10% and 30%) on PSD, and the transition to dependent scattering regimes. These extensions will further improve the applicability of Intralipid^®^ in biomedical optics.

## Supplementary Material

10.1117/1.JBO.31.1.015001.s01

## Data Availability

All relevant data can be found in the following Figshare repository: https://figshare.com/articles/dataset/Measured_data_and_processed_results/29522198.
