# Supplementary material for: Particle size distribution and concentration of Intralipid® 20%
Source: J Biomed Opt. 2026 Jan 23;31(1):015001. doi: 10.1117/1.JBO.31.1.015001 (PMC12828180; doi:10.1117/1.JBO.31.1.015001)
Supplement: Supplementary file 1 [file JBO_031_015001_SD001.pdf]

Supplemental information: Particle size distribution and concentration of Intralipid® 20 %

Mona Shahsavari,[a,b,c,d] Martine Kuiper,[a,b,e] Mendel Engelaer,[a,b,c,d] Martin Poinsinet de Sivry-Houle,[a,b,c,d] Ton G. van Leeuwen,[a,c,d] Edwin van der Pol[a,b,c,d]

[a]Amsterdam UMC, University of Amsterdam, Biomedical Engineering & Physics, Meibergdreef 9, Amsterdam, The Netherlands

[b]Amsterdam UMC, University of Amsterdam, Laboratory of Experimental Clinical Chemistry, Laboratory Specialized Diagnostics & Research, Department of Laboratory Medicine, Meibergdreef 9, Amsterdam, The Netherlands

[c]Amsterdam Cardiovascular Sciences, Atherosclerosis and Ischemic Syndromes, Amsterdam, The Netherlands

[d]Cancer Center Amsterdam, Imaging and Biomarkers, Amsterdam, The Netherlands

[e]VSL, National Metrology Institute, Delft, The Netherland

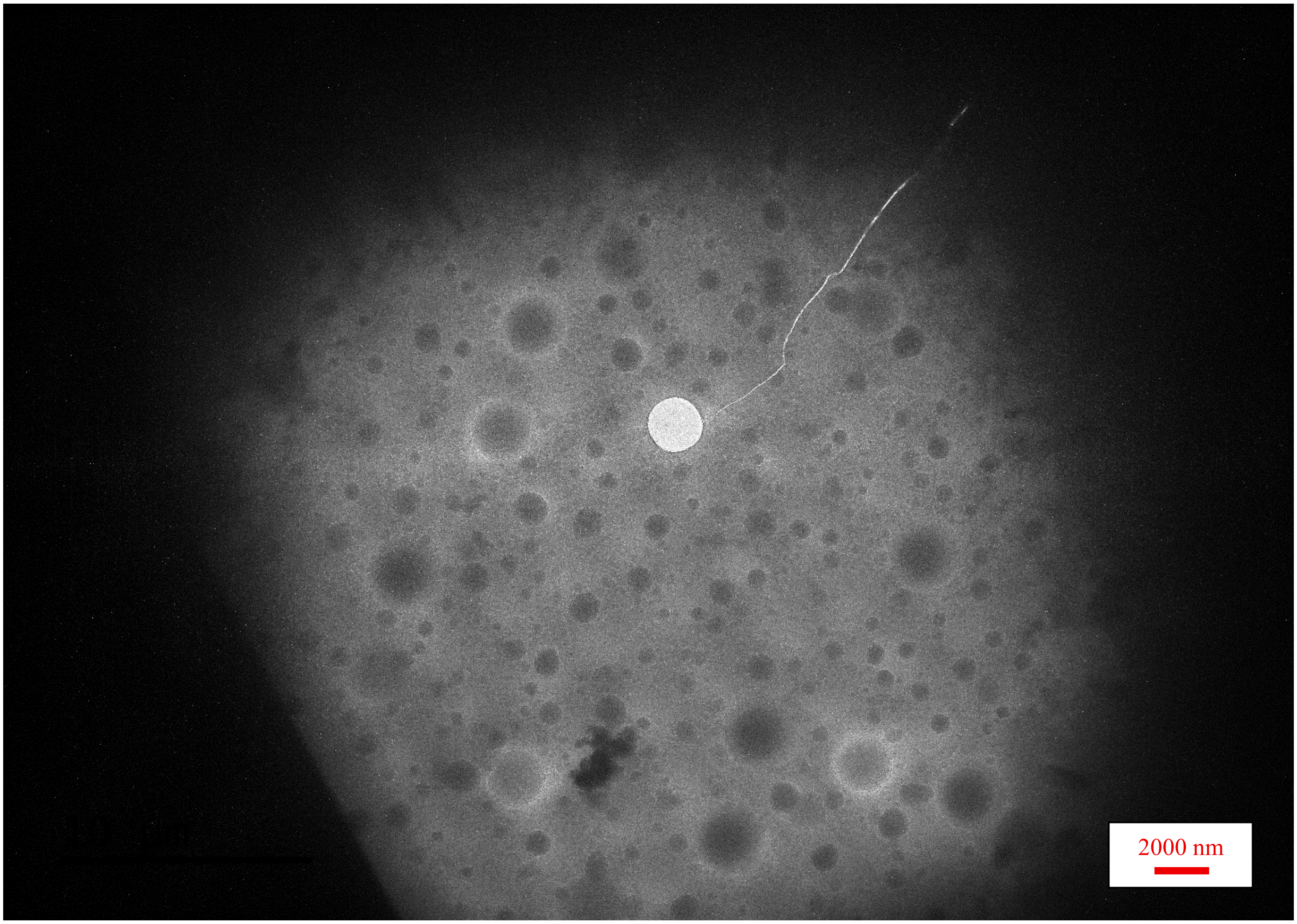

Fig. S1 Representative cryogenic electron microscopy (cryo-EM) image of 1 % (v/v) dilution of Intralipid® 20 % in Dulbecco's phosphate-buffered saline.

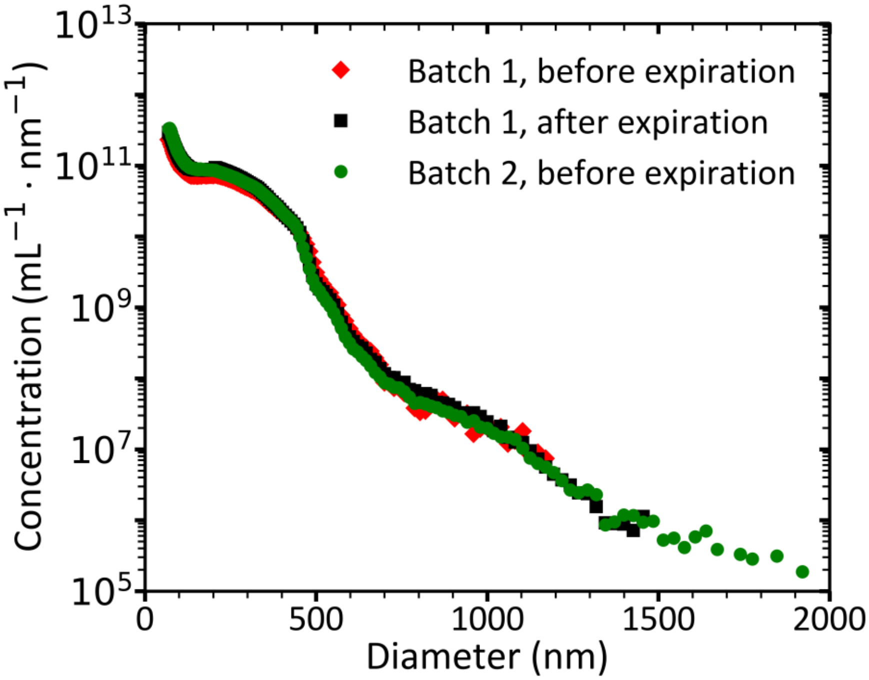

Fig. S2 Concentration versus particle diameter in stock Intralipid® 20 % measured using flow cytometer 1 (Northern Lights). Diamonds represent batch 1 of Intralipid® 20 % before expiration, squares represent batch 1 three months past its expiration date, and circles represent batch 2 before expiration. The particle size distributions (PSDs) are the combined results from two detector gain settings, as explained in Methods Sec. 2.4. For the gain-2000 measurements, the acquisition times were 2 minutes for all samples, while for the gain-10 measurements, acquisition times were 2 minutes for batch 1 before expiration, 10 minutes for batch 1 after expiration, and 30 minutes for batch 2. To report the stock concentration of Intralipid® 20 %, we multiplied the measured PSD of diluted samples, as described in the Methods Sec. 2.6, with the dilution factor. For calibrating flow cytometer results, particles in Intralipid® 20 % were modeled as soybean oil droplets (refractive index = 1.4927) in Dulbecco's phosphate-buffered saline (refractive index = 1.3446).
